# Enhancing pediatric fracture diagnostics with artificial intelligence: insights and recommendations

**DOI:** 10.1590/1806-9282.20251902

**Published:** 2026-08-10

**Authors:** Dong Li, Changjiang Zhang

**Affiliations:** 1 Zhengzhou University, Fifth Affiliated Hospital, Second Department of Orthopedics – Zhengzhou, China.

Dear Editor,

I read with interest the study by Kavak et al., which evaluates the efficacy of an artificial intelligence (AI) model in detecting pediatric appendicular fractures using deep learning technology^1^. This study demonstrates the substantial promise of AI in augmenting emergency department (ED) workflows and potentially reducing diagnostic errors in pediatric fracture cases, where misinterpretation is a frequent challenge due to anatomical variability and positioning issues.

While the results presented in this study are impressive, it is important to address several points that could further strengthen the applicability of AI in this domain. First, the AI model’s effectiveness, as evidenced by a sensitivity of 90% and specificity of 92%, is indeed noteworthy. However, variations in patient demographics, injury types, and imaging protocols across different healthcare settings could potentially impact the generalizability of these findings. Validation of this model in diverse clinical environments is recommended to confirm its robustness^2,3^.

Furthermore, the study indicates that AI-assisted diagnostics improved the sensitivity and accuracy of emergency doctors’ readings. However, given the potential for AI models to generate false positives (FP), as noted with the 7.6% FP rate in this study, it is crucial to implement training protocols that equip healthcare professionals to interpret AI suggestions judiciously, minimizing reliance on AI alone and maintaining clinical judgment^4,5^.

Lastly, although the study highlights the retrospective nature as a limitation, prospective studies could offer insights into realtime AI integration in clinical workflows and its impact on patient outcomes. AI has transformative potential in pediatric radiology, but longitudinal studies are needed to establish sustainable practices and optimize algorithm accuracy across patient populations.

In conclusion, Kavak et al.’s research provides a strong foundation for integrating AI into pediatric fracture diagnostics. With further refinements and expanded validations, AI could significantly enhance diagnostic accuracy and efficiency in ED settings, ultimately benefiting patient care.

## Data Availability

The datasets generated and/or analyzed during the current study are available from the corresponding author upon reasonable request.
